# *Diaporthe atlantica* enhances tomato drought tolerance by improving photosynthesis, nutrient uptake and enzymatic antioxidant response

**DOI:** 10.3389/fpls.2023.1118698

**Published:** 2023-02-01

**Authors:** Eric C. Pereira, Iñigo Zabalgogeazcoa, Juan B. Arellano, Unai Ugalde, Beatriz R. Vázquez de Aldana

**Affiliations:** ^1^ Plant-Microorganism Interactions Research Group, Institute of Natural Resources and Agrobiology of Salamanca, Consejo Superior de Investigaciones Científicas (IRNASA-CSIC), Salamanca, Spain; ^2^ Biofungitek Limited Society (S.L.) Parque Científico y Tecnológico de Bizkaia, Derio, Spain

**Keywords:** symbiosis, *diaporthe*, drought stress, fungi, antioxidant defense, nutrient uptake, proline, photosynthetic capacity

## Abstract

Functional symbiosis with fungal endophytes can help plants adapt to environmental stress. *Diaporthe atlantica* is one of the most abundant fungal taxa associated with roots of *Festuca rubra* subsp. *pruinosa*, a grass growing in sea cliffs. This study aimed to investigate the ability of a strain of this fungus to ameliorate the impact of drought stress on tomato plants. In a greenhouse experiment, tomato plants were inoculated with *Diaporthe atlantica* strain EB4 and exposed to two alternative water regimes: well-watered and drought stress. Several physiological and biochemical plant parameters were evaluated. Inoculation with *Diaporthe* promoted plant growth in both water treatments. A significant interactive effect of *Diaporthe*-inoculation and water-regime showed that symbiotic plants had higher photosynthetic capacity, water-use efficiency, nutrient uptake (N, P, K, Fe and Zn), and proline content under drought stress, but not under well-watered conditions. In addition, *Diaporthe* improved the enzymatic antioxidant response of plants under drought, through an induced mechanism, in which catalase activity was modulated and conferred protection against reactive oxygen species generation during stress. The results support that *Diaporthe atlantica* plays a positive role in the modulation of tomato plant responses to drought stress by combining various processes such as improving photosynthetic capacity, nutrient uptake, enzymatic antioxidant response and osmo-protectant accumulation. Thus, drought stress in tomato can be enhanced with symbiotic fungi.

## 1 Introduction

Drought is a multidimensional stress that causes a wide range of morphophysiological, biochemical and molecular modifications on plants, affecting their growth and development (Farooq et al., 2009; Chaves et al., 2011). At a cellular scale, a series of harmful perturbations in some central processes occur, including disorders in water homeostasis, perturbations in metabolic functions and hormonal imbalance. In addition, changes in chlorophyll synthesis, root differentiation, foliage development, stomatal movement, and water and mineral nutrition occur, leading to a decrease in plant yield and water use efficiency (Kapoor et al., 2020; Kaur et al., 2021). Drought also induces the generation of reactive oxygen species (ROS), which cause oxidative damage and disturb the cell redox regulatory functioning (Cruz de Carvalho, 2008; Impa et al., 2012).

To cope with water deficit, plants have developed mechanisms to capture more water from the soil or to minimize water loss *via* transpiration (Osakabe et al., 2014; Takahashi et al., 2020). Morphological changes such as an increase in root size for better exploring the soil and increasing surface absorption can occur (Hund et al., 2009). In response to drought stress, the stomatal closure reduces transpirational water loss, but also causes a decrease in both CO_2_ diffusion and photosynthetic carbon assimilation rate (Shahzad et al., 2016). The production of compatible organic solutes, such as proline, is another important mechanism to adapt to water deficit, contributing to osmotic adjustment, ROS detoxification, and stabilization of membrane, enzyme and protein structures (Farooq et al., 2009; Takahashi et al., 2020). In order to cope with oxidative stress under drought, plants also use antioxidant defense systems (Shahzad et al., 2016). The antioxidant apparatus helps to scavenge reactive oxygen species (ROS) and to regenerate ascorbate (AsA) using enzymatic antioxidants like catalase (CAT), ascorbate peroxidase (APX) or dehydroascorbate reductase (DHAR) (Koffler et al., 2014; Noctor et al., 2014; Laxa et al., 2019).

Tomato (*Solanum lycopersicum* L.) is one of the most important horticultural crops in the world. Its high sensitivity to water deficit has prompted different approaches for obtaining drought-resistant cultivars. The plant microbiome can have an important role in plant growth and stress tolerance, having applications related to crop production (Ray et al., 2020; Pozo et al., 2021).

*Diaporthe* is one of the most abundant fungal taxa associated with roots of *Festuca rubra* subsp. *pruinosa*, a grass growing in sea cliffs (Pereira et al., 2019). In this habitat, *F. rubra* grows in rock fissures where nutrient availability is scarce, and exposure to salinity is intense (Castroviejo, 2021). When inoculated in agricultural grasses, a *Diaporthe* strain ameliorated salt stress, increasing proline, nutrient uptake, and phytohormones, resulting in plant growth improvement (Toghueo et al., 2022). That fungal strain belongs to *Diaporthe atlantica*, a dominant species of the genus in *Festuca* roots (Toghueo et al., 2023). Symbiotic microorganisms from saline environments might benefit plants in their adaptation to drought stress (Rodriguez et al., 2008; Hosseyni Moghaddam et al., 2021). Plant responses to drought and salinity have much in common because both conditions induce osmotic stress and oxidative damage in an early stage, which leads to a decrease in growth, stomatal aperture, and a deficit in nutrients (Forni et al., 2017; Ma et al., 2020). Therefore, plant adaptation to both stresses could be mediated by similar mechanisms involving plant responses such as growth attenuation, accumulation of compatible solutes as proline, increased levels of antioxidants and protective proteins, suppression of energy-consuming pathways and gene expression regulation (Bartels and Sunkar, 2005; Munns, 2011).

Thus, the main objective of this work was to evaluate the ability of a *Diaporthe atlantica* strain isolated from *Festuca rubra* subsp. *pruinosa* to improve the growth and drought tolerance of tomato plants. For this purpose, the changes of tomato plants in physiological and biochemical parameters such as chlorophyll, gas exchange, mineral elements, proline, antioxidant enzyme activities and antioxidant capacity were evaluated.

## 2 Materials and methods

### 2.1 Fungal material

The *Diaporthe* strain EB4 was originally isolated from surface-disinfected roots of an asymptomatic plant of *Festuca rubra* subsp. *pruinosa*, collected in a natural population on the northern coast of Galicia, Spain (Pereira et al., 2019). This strain belongs to *Diaporthe atlantica*, a newly described species (Toghueo et al., 2023).

Most *Diaporthe atlantica* strains, including EB4, do not sporulate on laboratory media (Toghueo et al., 2023), for this reason, fungal mycelium was used as inoculum. To produce *Diaporthe* EB4 mycelial inoculum, 30 g of sugar beet pulp pellet mixed with 9.0 g CaCO_3_, 4.5 g CaSO_4_ and hydrated with 60 ml of water were autoclaved in wide-mouth glass bottles for 30 minutes at 121°C (Vázquez de Aldana et al., 2020). Each bottle of sugar beet pulp substrate was inoculated with four plugs of mycelium from a potato dextrose agar (PDA) culture and incubated at room temperature (20-22°C) for four weeks.

### 2.2 Experimental design

To determine the effect of *Diaporthe* inoculation on tomato plants under drought stress, a bioassay with two variables was designed: *Diaporthe* inoculation (inoculated or uninoculated plants) and water treatment (well-watered and drought stress). For each of the four treatments, ten replicates were considered. To inoculate plants, seeds of tomato cv. Marmande were sown in a plastic tray containing a substrate composed of seven parts of peat and perlite (1:1 v/v), previously treated at 80°C for 24 h, and one part of *Diaporthe* EB4 inoculum. Uninoculated plants were obtained from seeds sown in a tray containing only the peat and perlite mixture. Ten-day-old seedlings were individually transplanted to 300-ml plastic plots containing the heat-treated substrate with or without inoculum for the inoculated and uninoculated seedlings, respectively.

During the first week, all plants were exposed to a well-watered regime. After this period of adaptation, two watering treatments were applied for five weeks: a well-watered, and a drought stress regime. In the well-watered regime, plants were watered three times per week at 100% of the water holding capacity. In the drought stress treatment, plants were watered three times per week at 10% of the water holding capacity of the soil. To avoid plant death under drought stress, these plants were watered once at 100% of the water holding capacity three weeks after the drought treatment was initiated.

Five weeks after the start of the watering treatment, all plants were harvested. Three leaves from the same branch were collected from each plant and immediately immersed in liquid nitrogen and kept at −80°C for antioxidant enzyme analysis. Then, each plant was separated into leaves, stems, and roots and lyophilized to measure dry weight and for chemical analyses.

### 2.3 Detection of *Diaporthe* in inoculated plants

The presence of *Diaporthe* in inoculated plants was diagnosed by light microscopy in root samples collected at harvest time. Fresh root fragments were cleared in 5% KOH at 90°C for 15 min, neutralized with approximately three volumes of 1% HCl at 20°C overnight, stained with trypan blue (Berthelot et al., 2016), and visualized.

### 2.4 Measurements of plant physiological and biochemical parameters

#### 2.4.1 Photosynthetic parameters

The chlorophyll content was determined 24 h before plant harvesting by means of a leaf-clip sensor (Dualex Force, Orsay, France). In each plant, three leaves of the third branch from the top were selected, and the average chlorophyll content was obtained from three measurements taken at the central position of each leaf.

The gas exchange measurements at 400 ppm CO_2_, including stomatal conductance, CO_2_ assimilation rate, and water use efficiency (WUE) were obtained from leaves of the third branch from the top of four randomly replicate plants per treatment, making use of a CIRAS-3 portable gas exchange system (PP-Systems, Amesbury, MA, USA) 24 h before plant harvesting. The leaves were pressed between the upper and lower gaskets of the leaf cuvette head of CIRAS-3 and pre-acclimated for 15−20 min.

#### 2.4.2 Analysis of mineral element content

The concentration of mineral elements (N, P, K, Ca, Fe, S and Zn) was analyzed in five replicates of leaf samples. For that purpose, freeze-dried and ground samples were calcined at 450°C for 8 h, and ashes were dissolved in HCl : HNO_3_:H_2_O (1:1:8). Then, P, K, Ca, Fe, S and Zn contents were determined by inductively coupled plasma atomic emission spectroscopy (ICP-OES) in a Varian 720-ES spectrometer (Agilent, USA). Carbon and Nitrogen contents were analyzed by the Dumas combustion method in a C-N analyzer (Leco CHN-628, USA).

#### 2.4.3 Antioxidant enzyme determination

At harvest time, the third leaf from three different branches of the same plant were pooled for antioxidant enzyme activity assays. Samples of fresh leaves previously stored at −80°C were ground with liquid nitrogen and kept at −80°C until the measurement of the antioxidant enzyme activities. The antioxidant activities of catalase (CAT), ascorbate peroxidase (APX), and dehydroascorbate reductase (DHAR) were measured in leaf samples of four plant replicates per treatment following the methods described below by Bendou et al. (2022) and Pérez-López et al. (2009). APX was selected as a representative peroxidase activity enzyme because it belongs to the ascorbate-glutathione cycle, it is very sensitive to stress conditions, and it is well established that APX also regulates redox signaling pathways in normal plant development (Caverzan et al., 2012). A 96-well microplate reader FLUOstart^®^ Omega (BMG Labtech, Ostenberg, Germany) was used for all the spectrophotometric methods.

For CAT activity, 40 mg of the ground samples were mixed with 0.5 ml of 50 mM Tris-HCl (pH= 7.8), 0.1 mM EDTA, 0.2% (v/v) Triton X−100, 1 mM phenylmethylsulfonyl fluoride (PMSF) and 2 mM dithiothreitol and beaten with glass beads for 1 min. The homogenates were filtered through a layer of muslin and gel-filtered over MicroSpin G25 columns (Amersham Biosciences, Sweden) equilibrated with 50 mM Tris−HCl (pH= 7.8), 0.1 mM EDTA and 0.2% (v/v) Triton X−100. CAT activity was measured spectrophotometrically by monitoring the disappearance of H_2_O_2_ at 240 nm in a reaction mixture of a final volume of 300 μl containing 50 mM potassium phosphate buffer (pH= 7.0), 25 mM H_2_O_2_ and 5 μl of the filtered supernatant.

The homogenizing medium for DHAR analysis consisted of 50 mM potassium phosphate (pH= 7.8), 0.1 mM EDTA, 0.2% (v/v) Triton X−100, 2 mM AsA, 5 mM cysteine, 0.1 mM PMSF and 1% (w/v) poly(vinylpolypyrrolidone). An amount of 40 mg of ground samples were incubated with 0.5 ml of the homogenizing buffer for 10 min at 6−8°C, filtered through a layer of muslin and centrifuged at 16,100 *g* for 15 min. DHAR activity was determined by monitoring AsA formation *via* dehydroascorbate (DHA) reduction at 265 nm. Briefly, the final volume of the assay mixture was 300 μl, and contained 2.5 mM glutathione (GSH), 0.1 mM EDTA, 50 mM potassium phosphate (pH= 6.6) and 10 μl of supernatant. The reaction was initiated by adding 10 μl of 0.2 mM DHA to the reaction mixture. The reaction rate was corrected for the non-enzymatic reduction of DHA by GSH.

For the APX activity, the ground samples were homogenized as in the previous paragraph. APX activity was analyzed by measuring the oxidation of AsA at 290 nm. Briefly, a volume of 290 μl of reaction mixture containing 0.8 mM AsA and 50 mM HEPES (pH= 7.6) was mixed with 10 μl of the supernatant. The oxidation rate of AsA measured as the decline in absorbance at 290 nm was estimated 1−6 min after starting the reaction with the addition of H_2_O_2_ at a final concentration of 1.2 mM. Corrections were made for the non-enzymatic oxidation of ascorbate by H_2_O_2_ and for the oxidation of ascorbate in the absence of H_2_O_2_.

The measurement of the CAT, APX and DHAR activities were carried out 25°C and protein content in the supernatant was measured according to the Bradford method (Bradford, 1976).

#### 2.4.4 Ferric reducing antioxidant potential assay

The total antioxidant capacity was determined in leaves of five replicates of each treatment using the ferric ion reducing antioxidant power (FRAP) method (Benzie and Strain, 1996). This method is based on the reduction of the colorless [Fe(III)−,4,6-tri(2-pyridyl)-*s*-triazine)_2_]^3+^ complex, abbreviated as Fe(III)-TPTZ, to the blue-colored Fe(II)-TPTZ complex, formed by the action of electron donating antioxidants at low pH. The FRAP reagent was prepared by mixing 300 mM acetate buffer (pH=3.6), a solution of 10 mM TPTZ in 40 mM HCl, and 20.35 mM FeCl_3_ at a ratio of 10:1:1 (v/v/v). Five mg of each plant sample were extracted in 700 µl of 50% aqueous acetone for 30 min in an ultrasound bath at 8°C. The mixture was centrifuged and transferred to a 96-well plate where 8 µl of the sample, 8 µl of phosphate buffer saline, and 200 µl of FRAP reagent were added to each well. The absorbance was measured at 593 nm after 30-min incubation in a microplate reader FLUOStar Omega (BMG Labtech, Ostenberg, Germany). A standard curve was prepared using different concentrations of 6-hydroxy-2,5,7,8-tetramethylchroman-2-carboxylic acid (Trolox). The results were expressed as μmol trolox equivalent/g dry weight.

#### 2.4.5 Total phenolic compounds content

The content of total phenolic compounds in leaf samples (five replicates of each treatment) was determined spectrophotometrically according to the Folin-Ciocalteu method (Ainsworth and Gillespie, 2007). An aliquot of 100 µl of 50% aqueous acetone extract of each sample, prepared as previously described for the FRAP assay was mixed with 500 µl of Folin-Ciocalteu reagent (Scharlab Chemie S.A.). After 5 min, a volume of 400 μl of a 700 mM Na_2_CO_3_ solution was added. The mixture was incubated for 60 min and the absorbance at 765 nm was measured in a 96-well plate in a microplate reader FLUOStar Omega (BMG Labtech, Ostenberg, Germany). Gallic acid was used as a reference standard, and the results were expressed as μmol gallic acid equivalent/g dry weight.

#### 2.4.6 Proline content

Proline content was quantified in leaves of five plant replicates per treatment using the spectrophotometric method described by Shabnam et al. (2016), adapted to 96-well plates in our laboratory. Approximately 15 mg of freeze-dried and ground plant material were homogenized in 500 µl of 3% aqueous sulfosalicylic acid and kept for 10 min in ice. The mixture was centrifuged at 10°C and 16,000 *g* for 10 min and the supernatant was mixed with 250 μl of glacial acetic and 500 µl of ninhydrin reagent. Then, the mixture was heated at 99°C for 40 min and immediately cooled with ice. The mixture was centrifuged and an aliquot of 200 µl was transferred to a 96-well plate where the absorbance was measured at 513 nm in a microplate reader FLUOStar Omega (BMG Labtech, Ostenberg, Germany). L-proline (Acrós Organics) was used as a standard for quantification.

### 2.5 Statistical analyses

The data were evaluated for statistical assumptions of the ANOVA using the Shapiro-Wilk normality test and Levene´s equal variance test. The effect of *Diaporthe* inoculation and water treatment on plant parameters were analyzed with a two-way ANOVA. Differences between treatment means were evaluated by Tukey’s test. All the statistical analyses were performed by means of Sigma-Plot 14.5.

## 3 Results

### 3.1 Detection of *Diaporthe* in inoculated plants

Fungal structures were not observed by light microscopy in the roots of inoculated plants. Therefore, it appears that the association of *Diaporthe* EB4 with tomato plants may be rhizospheric and not endophytic.

No visual disease symptoms were observed on roots or leaves of plants inoculated with *Diaporthe*, regardless of the water regime. This indicates that this *Diaporthe* strain is not pathogenic to tomato plants.

### 3.2 Effect of *Diaporthe* and water regime on plant biomass production

**Figure 1:**
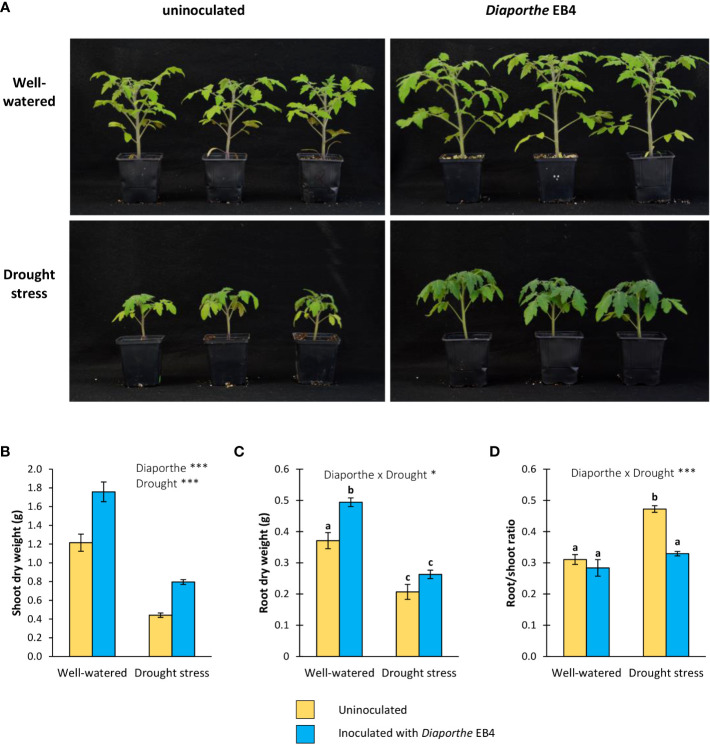
**(A)** Six-week-old tomato plants uninoculated (yellow) or inoculated with *Diaporthe* strain EB4 (blue), with two different water treatments (well-watered or drought stress), **(B)** shoot biomass, **(C)** root biomass, and **(D)** root/shoot ratio. Different letters indicate different means (Tukey *p*<0.05) for the [*Diaporthe* inoculation × Drought] interaction. Values are means +SE (n=10). Level of significance: *p < 0.05; ***p < 0.001.

In terms of dry weight, both inoculation and water treatment significantly affected the shoot growth of tomato plants. However, the interaction of both factors was not significant (**Figure 1**; **Table 1**). The shoot biomass increased in inoculated plants regardless of drought stress. Compared to uninoculated plants, *Diaporthe* increased the shoot biomass by 45% in well-watered plants, and by 80% under drought. Compared to the well-watered treatment, drought significantly reduced the shoot biomass by 58% (**Figures 1A, B**).

**Table 1:** Results of two-way analysis of variance showing the effect of inoculation with *Diaporthe* EB4, water treatment and their interaction on tomato.

	*Diaporthe* inoculation		Water treatment		*Diaporthe* × watering	
	F	P	F	P	F	P
Shoot dry weight	39.60	**<0.001**	146.1	**<0.001**	1.738	0.196
Root dry weight	14.47	**<0.001**	113.6	**<0.001**	5.724	**0.022**
root/shoot ratio	25.91	**<0.001**	38.58	**<0.001**	12.12	**0.001**
Chlorophyll content	122.9	**<0.001**	18.67	**<0.001**	33.92	**<0.001**
Stomatal conductance	16.21	**0.002**	0.574	0.463	1.007	0.335
CO_2_ assimilation	26.69	**<0.001**	0.462	0.510	7.023	**0.021**
WUE	8.332	**0.014**	24.97	**<0.001**	23.94	**<0.001**
N	26.47	**<0.001**	81.15	**<0.001**	9.942	**0.006**
P	16.51	**<0.001**	45.90	**<0.001**	6.105	**0.025**
K	0.704	**0.414**	156.0	**<0.001**	20.46	**<0.001**
Ca	13.99	**0.002**	0.439	0.517	1.125	0.305
Fe	1.172	0.295	56.13	**<0.001**	18.24	**<0.001**
S	240.4	**<0.001**	84.97	**<0.001**	43.72	**<0.001**
Zn	37.43	**<0.001**	76.33	**<0.001**	26.96	**<0.001**
C	1.403	0.253	0.352	0.561	0.622	0.442
CAT	9.126	**0.011**	48.80	**<0.001**	8.816	**0.012**
DHAR	4.880	**0.047**	9.241	**0.002**	2.207	0.163
APX	0.095	0.763	40.10	**<0.001**	3.366	0.091
Antioxidant capacity	1.472	0.243	25.27	**<0.001**	16.59	**<0.001**
Phenolic compounds	2.107	0.166	1.168	0.296	4.555	0.057
Proline	6.297	**0.023**	11.97	**0.030**	4.662	**0.046**

Numbers in bold indicate that the factor significantly affects the variable.

For the root biomass, a significant effect of inoculation, water treatment, and their interaction was detected (**Figure 1C**; **Table 1**). The root biomass increased in inoculated compared to uninoculated plants in the well-watered treatment (33%), but this difference was not significant under drought stress (**Figure 1C**). The root:shoot ratio increased in uninoculated repect to inoculated plants under drought, but the difference in the well-watered treatment was not significant (**Figure 1D**).

### 3.3 Effect of *Diaporthe* and water regime on photosynthesis activity and WUE

**Figure 2:**
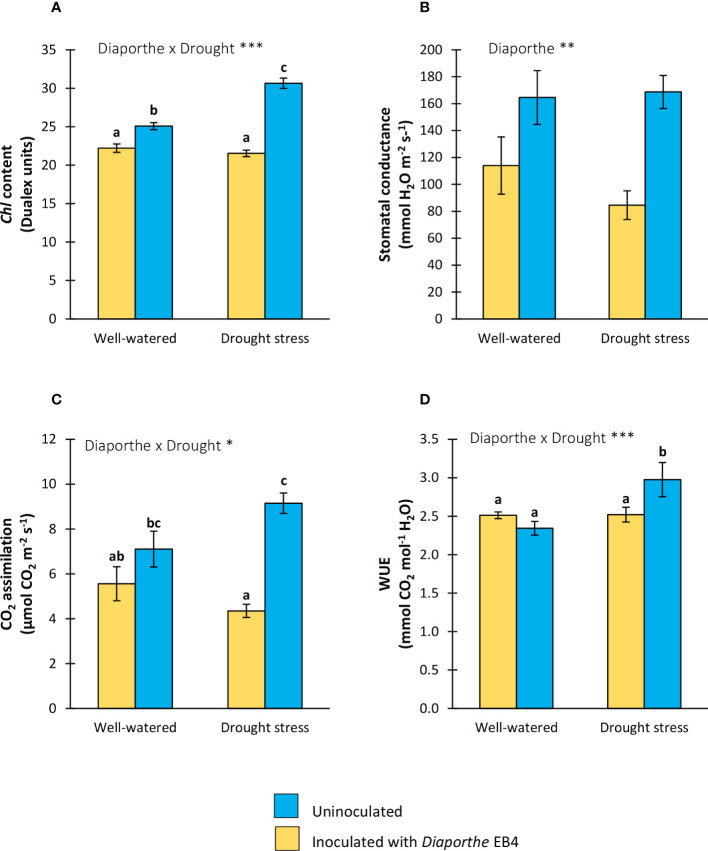
**(A)** Chlorophyll content, **(B)** stomatal conductance, **(C)** CO_2_ assimilation rate, and **(D)** water use efficiency (WUE), of tomato plants uninoculated (yellow) or inoculated with *Diaporthe* EB4 (blue), with two different water treatments (well-watered or drought stress). Different letters indicate different means (Tukey *p* < 0.05) for the [*Diaporthe* inoculation × Drought] interaction. Values are means +SE (n=5). Level of significance:*p < 0.05; **p < 0.01; ***p < 0.001.

A significant effect of *Diaporthe*, water treatment, and their interaction was detected on the chlorophyll content (**Table 1**). Compared to uninoculated plants, the chlorophyll content increased significantly with *Diaporthe* inoculation, and this increase was larger under drought stress than in well-watered plants (**Figure 2A**). The inoculation with *Diaporthe* significantly increased the stomatal conductance regardless of the water regime (**Figure 2B**; **Table 1**).

A significant effect of *Diaporthe* and its interaction with water treatment was detected on the CO_2_ assimilation rate (**Table 1**). Compared to uninoculated, this parameter increased in inoculated plants under drought stress, but the difference in well-watered plants was not significant (**Figure 2C**). In parallel to these results, the WUE increased in inoculated plants compared to uninoculated under drought stress, but such a difference was not significant in well-watered plants (**Figure 2D**).

### 3.4 Effect of *Diaporthe* and water regime on mineral elements content

**Figure 3:**
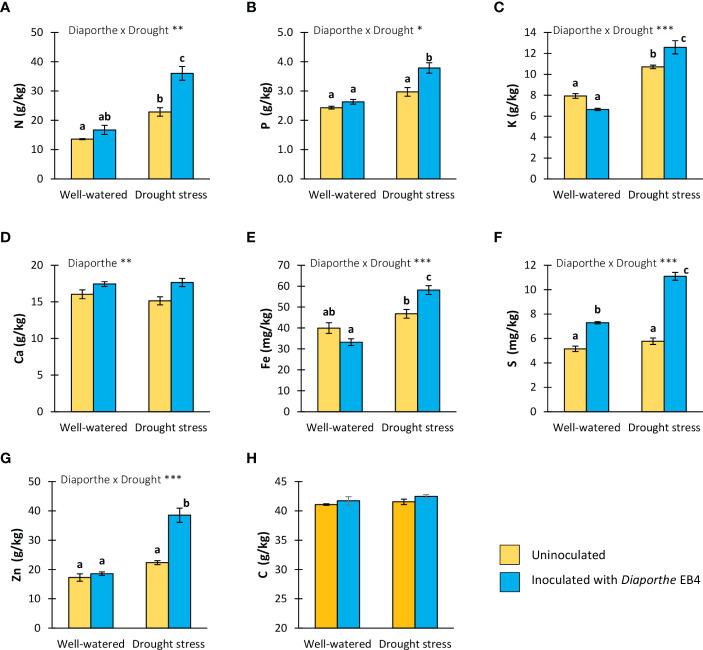
**(A)** Total nitrogen, **(B)** phosphorus, **(C)** potassium, **(D)** calcium, **(E)** iron, **(F)** sulphur, **(G)** zinc and **(H)** total carbon contents in tomato plants uninoculated (yellow) or inoculated with *Diaporthe* EB4 (blue), with two different water treatments (well-watered or drought stress). Different letters indicate different means (Tukey *p*<0.05) for the [*Diaporthe* inoculation × Drought] interaction. Values are means +SE (n=5). Level of significance: *p < 0.05; **p < 0.01; ***p < 0.001.

The N, P, K, Fe, S and Zn content was significantly affected by the *Diaporthe* × water treatment interaction (**Table 1**). Compared to uninoculated, the concentration of N, P, K, Fe and Zn increased significantly in inoculated plants under drought stress, but differences in the well-watered treatment were not significant (**Figure 3**). The S content increased due to *Diaporthe* in both well-watered and drought treatments (**Figure 3F**). The Ca concentration was only significantly affected by *Diaporthe* inoculation, increasing in inoculated plants regardless of water regime (**Figure 3D**). The total C content was not significantly affected by any factor or their interaction (**Figure 3H**, **Table 1**).

### 3.5 Effect of *Diaporthe* and water regime on biochemical plant parameters

#### 3.5.1 Antioxidant enzyme activity

**Figure 4:**
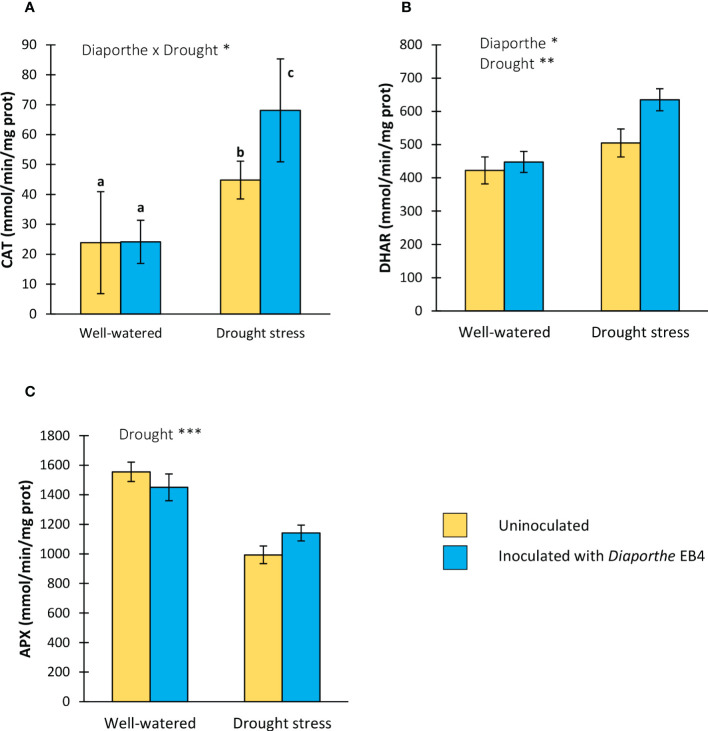
Activity of the antioxidant enzymes **(A)** catalase (CAT), **(B)** dehydroascorbate reductase (DHAR), and **(C)** ascorbate peroxidase (APX) of tomato plants uninoculated (yellow) or inoculated with *Diaporthe* EB4 (blue), with two different water treatments (well-watered or drought stress). Different letters indicate different means (Tukey *p* < 0.05) for the [*Diaporthe* inoculation × Drought] interaction. Values are means +SE (n=5). Level of significance: *p < 0.05; **p < 0.01; ***p < 0.001.

A significant effect of *Diaporthe*-inoculation, water treatment, and their interaction was detected on the activity of catalase (CAT) (**Table 1**). The CAT activity increased with *Diaporthe* inoculation, but only when plants were subjected to drought stress (**Figure 4A**). DHAR activity was affected by *Diaporthe* inoculation and drought stress, but not by their interaction (**Table 1**). The DHAR activity increased under drought stress regardless of inoculation, and in inoculated plants regardless of water treatment (**Figure 4B**). The APX activity was significantly lower in plants under drought stress regardless of inoculation (**Figure 4C**).

#### 3.5.2 Antioxidant capacity and phenolic compounds content

**Figure 5:**
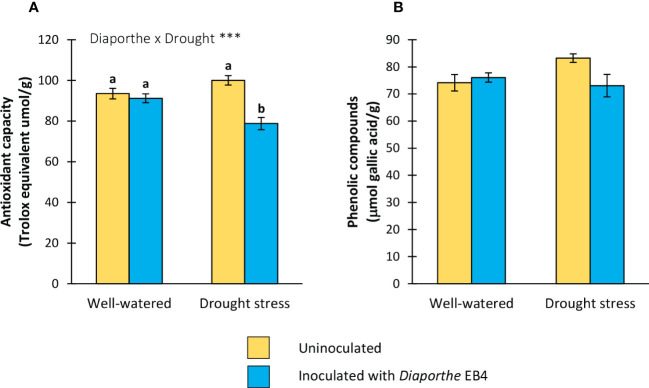
**(A)** Antioxidant capacity, and **(B)** total phenolic compounds content of tomato plants uninoculated (yellow) or inoculated with *Diaporthe* EB4 (blue), with two different water treatments (well-watered or drought stress). Different letters indicate different means (Tukey *p*<0.05) for the [*Diaporthe* inoculation × Drought] interaction. Values are means ± SE (n=5). Level of significance: ***p < 0.001.

A significant effect of the inoculation × water treatment interaction was detected on the antioxidant capacity (**Table 1**). Compared to uninoculated, this parameter decreased in *Diaporthe*-inoculated plants, but only under drought stress (**Figure 5A**). The phenolic compound content was not significantly affected by any factor (**Figure 5B**).

#### 3.5.3 Proline content

**Figure 6:**
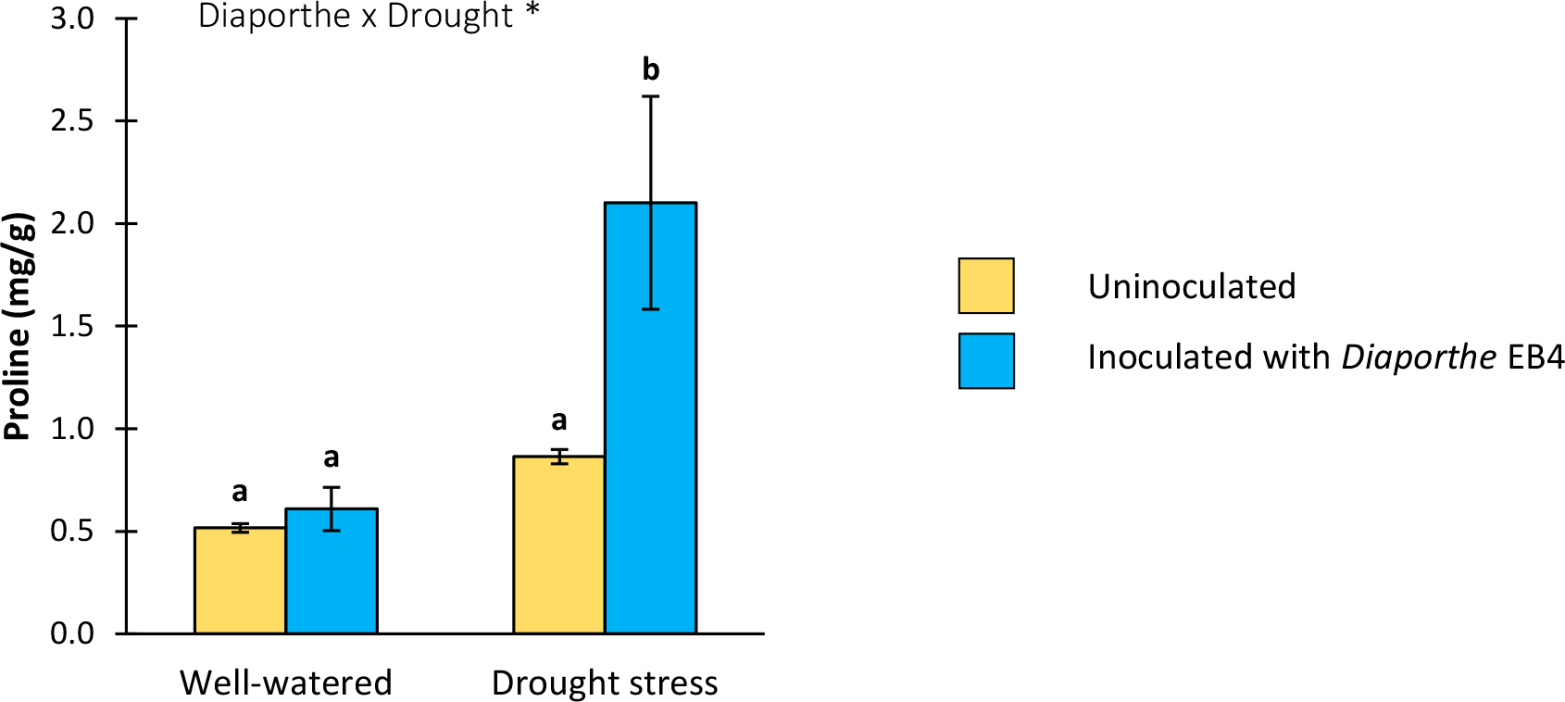
Proline content in leaves of tomato plants uninoculated (yellow) or inoculated with *Diaporthe* EB4 (blue), with two different water treatments (well-watered or drought stress). Different letters indicate different means (Tukey *p*<0.05) for the [*Diaporthe* inoculation × Drought] interaction. Values are means +SE (n=5). Level of significance: *p < 0.05.

A significant effect of *Diaporthe* inoculation, drought stress, and their interaction was detected on the proline content (**Table 1**). Compared to uninoculated plants, this osmolyte increased significantly in inoculated plants under drought stress; however, *Diaporthe* did not change the proline content in well-watered plants (**Figure 6**).

## 4 Discussion

*Diaporthe* species are one of the most abundant components of the culturable fungal microbiome of *Festuca rubra* subsp. *pruinosa* roots (Pereira et al., 2019). These plants grow in an habitat where exposure to salinity and limited soil nutrients are characteristic. *Diaporthe atlantica* strain EB4, isolated from roots of *Festuca rubra* subsp. *pruinosa*, was recently shown to improve plant growth and alleviate salt stress in two agricultural grasses: tritordeum and perennial ryegrass (Toghueo et al., 2022). This finding prompted us to analyze new symbiotic systems in which we could investigate the potential benefits of *Diaporthe* EB4 with non-gramineous agricultural plants of economic relevance such as tomato.

The genus *Diaporthe* includes pathogenic and endophytic species (Gomes et al., 2013). Tomato plants inoculated with *Diaporthe* EB4 exhibited an apparently healthy phenotype with no obvious disease symptoms. In addition, we did not observe by light microscopy any fungal structures inside the plant root tissues. This led us to conclude that *Diaporthe* EB4 should hold a non-pathogenic, epiphytic association with tomato plants, and moved forward to run experiments in which tomato plants were challenged with drought stress.

Although there was no experimental evidence for an endophytic association between *Diaporthe* EB4 and tomato, inoculated plants performed better than uninoculated plants, showing more biomass under both water regimes. Plants under drought stress showed evident changes in morphology, including lower plant biomass, smaller height, lower number of branches and reduced leaf area, all detrimental characteristics usually associated with slower plant cell expansion and division rates (Jaleel et al., 2009). This proved that a beneficial symbiotic association between *Diaporthe* EB4 and tomato plants occurred. Some plant-fungal symbiotic associations are known to enhance water retention and nutrient absorption, which, in turn, increase photosynthesis and production of stored material resulting in better root and shoot biomass (Li et al., 2019; Sarkar et al., 2021).

Previously it was observed that *Diaporthe* EB4 caused an enhancement of the content of abscisic (ABA) and indole-acetic acid (IAA) in leaves of tritordeum under salt stress, accompanied by an increase in the root and shoot biomass (Toghueo et al., 2022). In addition, *Diaporthe* EB4 cultures produced extracellular IAA (Toghueo et al., 2022). ABA and IAA are well known for their roles in maintaining water retention capacity and hydraulic properties in plants under drought, and modulating changes in root morphology (Tiwari et al., 2017; Saleem et al., 2018). Thus, *Diaporthe* EB4 could induce the formation of fine roots under drought stress, increasing the root-soil contact, and improving nutrient and water uptake. Recently, *Diaporthe masirevici* was demonstrated to have a positive effect on plant development by enhancing IAA production and phosphate solubilization (da Silva Santos et al., 2022).

**Figure 7:**
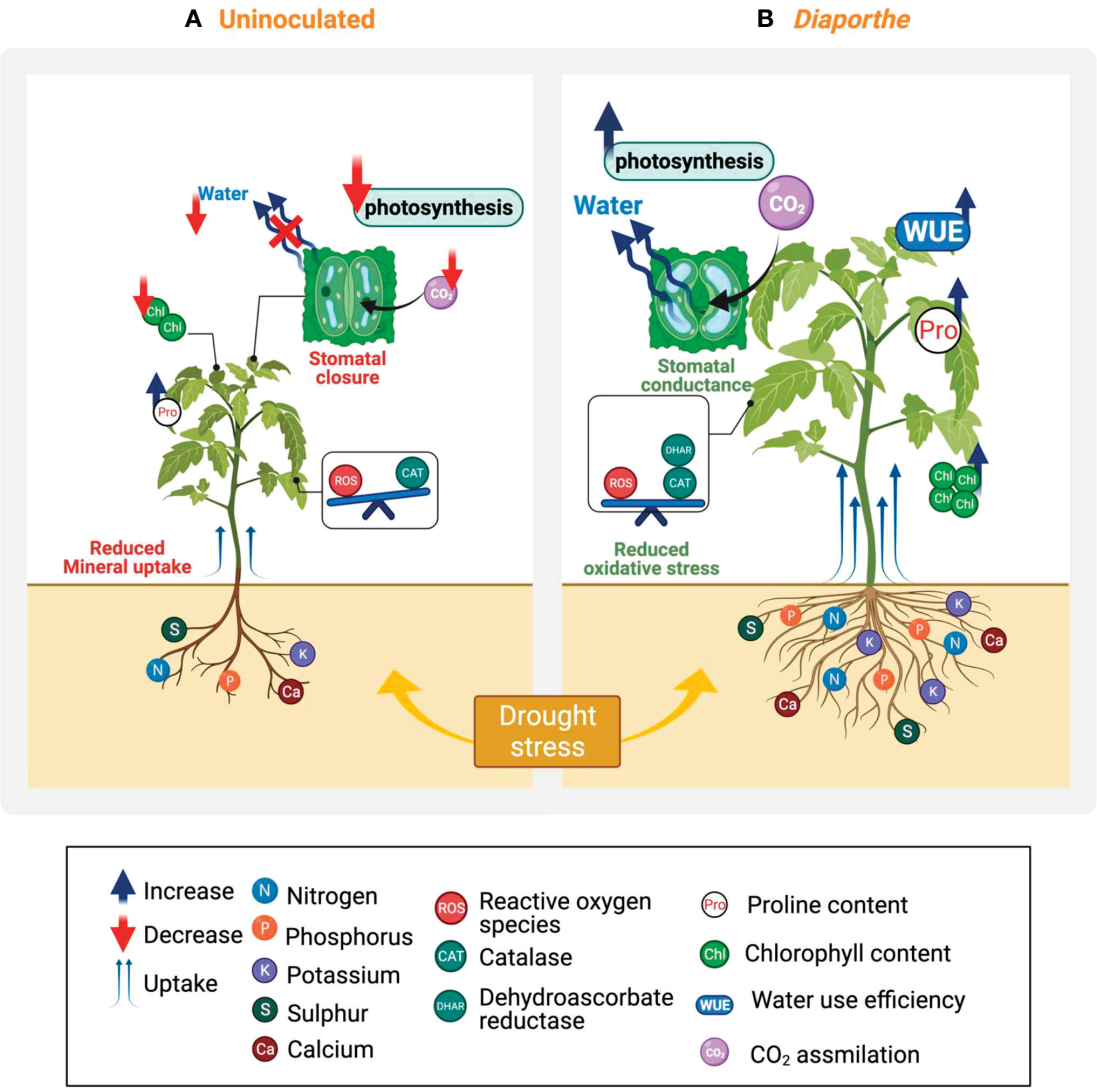
Overview of the effect of drought stress in uninoculated and *Diaporthe*-inoculated tomato plants. **(A)** Drought stress had a deleterious effect on tomato plant growth and biomass production. This biomass reduction can be associated with a reduction in photosynthetic activity caused by a reduction in stomatal conductance and consequently in the CO_2_ assimilation rate, and also by a decline in the chlorophyll content. The stomatal closure decreased the water movement on the plant which can be also associated with a decrease in the mineral uptake. In response to drought stress, the activity of CAT and proline content increased to reduce oxidative damage and for an osmotic counterbalance, however, this increase does not seem to be enough to alleviate the negative effect. **(B)** *Diaporthe* significantly mitigated the harmful impact of drought stress through combined mechanisms, which include an increase in the chlorophyll content, an optimal stomatal conductance that facilitates the CO_2_ assimilation, and a greater WUE, indicating the plant maintains its stomata open and subsequently preserves an optimal photosynthesis activity. *Diaporthe* stimulated the increase of antioxidant defense system, e.g., CAT and DHAR, suggesting a reduction of the oxidative stress caused by water limitations; significantly enhanced the proline content that can participate in the osmotic adjustment or in the structure protection, and increased the mineral uptake. All together favor plant growth under drought stress.

In this study, *Diaporthe* EB4 stimulated soil uptake and mobilization to the plant shoot of several macro- and micronutrients (N, P, K, Ca and S, and Fe and Zn) with essential roles in plant development, biosynthesis of photosynthetic pigments and proteins, photosynthesis and hormonal water regulation (Ahmad and Abdin, 2000; Peng et al., 2007; Hänsch and Mendel, 2009). The increase in the content of the above mineral nutrients, related to an increase in shoot biomass, was particularly significant in *Diaporthe–*inoculated plants under drought conditions. *Diaporthe* EB4 could suppress, at least in part, the negative effect of drought stress on plant biomass through a more efficient system of absorption of nutrients (**Figure 7**). The fact that the inoculated plants under drought had an unexpectedly higher mineral content than those under well-watered conditions was attributed to a dilution effect on the mineral nutrient content in inoculated plants under well-watered (and more favorable growth) conditions, in which the C metabolism was not downregulated and the partitioning of C towards structural components was not restricted as observed under drought stress (Ghaffari et al., 2019). In our study, the increase in biomass of *Diaporthe*-inoculated tomato plants seems to be conveyed by hormone mediated root structural changes leading to improved mineral uptake and water retention.

The decrease in plant growth caused by drought is also associated with the downregulation of photosynthesis (Parkash and Singh, 2020). In the present study, drought stress caused an evident reduction in the stomatal conductance and the CO_2_ assimilation rate of leaves in uninoculated plants, thereby limiting the synthesis and sink distribution of photosynthates. However, no significant changes in chlorophyll content were observed in uninoculated plants between drought and well-watered conditions, suggesting that, although there was a prominent decline in shoot biomass, the photosynthetic apparatus did not sustain severe photodamage. Interestingly, *Diaporthe* enhanced the chlorophyll content and the CO_2_ assimilation rate under both water treatments. The net CO_2_ assimilation rate in inoculated plants under drought was highest and correlated with the highest content of N and chlorophyll in leaves. This information can be used to predict that the maximum carboxylation rate by Rubisco (Vcmax) should also be the highest in inoculated plants under drought stress (Wang et al., 2021). Similar effects have been reported in other symbiotic systems. For example, *Diaporthe liquidambari* improved N accumulation in rice (Yang et al., 2014; Yang et al., 2015) and an increase in chlorophyll content was observed in *Trichoderma*–inoculated *Theobroma cacao* and *Neotyphodium–*inoculated *Elymus dahuricus* under drought stress (Zhang and Nan, 2007; Bae et al., 2009), whereas an enhancement of net CO_2_ assimilation was reported in *Neotyphodium*–infected tall fescue (Newman et al., 2003). Likewise, an improved adaptation to drought stress was observed in barley inoculated with *Piriformospora indica* as a result of enhanced activity of key enzymes of the N metabolism and a better distribution of N in the plant (Ghaffari et al., 2019).

*Diaporthe* EB4 was shown to increase the IAA content of *Lolium perenne* and tritordeum plants exposed to salt stress (Toghueo et al., 2022), and exogenous application of IAA was reported to increase the chlorophyll content in maize exposed to salt stress, and to stimulate stomatal aperture due to improved concentration of K in cells (Kaya et al., 2013). In this regard, the accumulation of macronutrients like K in leaves, together with an increase in IAA, seems to optimize leaf CO_2_ assimilation and water use. In our study, *Diaporthe-*inoculated plants under drought stress exhibited the greatest WUE, even though the stomatal conductance increased. In contrast, plants of *Lolium arundinaceum* symbiotic and non-symbiotic with *Epichloë coenophialum* (growing in the aboveground plant parts) held similar transpiration rates (Swarthout et al., 2009). In our study, the improvement of the relationship between the assimilated CO_2_ molecules and the loss of H_2_O molecules by transpiration was mainly attributed to a higher Rubisco activity (higher Vcmax) in the leaves of inoculated plants under drought, instead of a decrease in stomatal opening. Indeed, water movement through the xylem vessels could be enhanced in inoculated plants under drought stress because of the higher soil uptake of K by *Diaporthe*–colonized roots. Therefore, *Diaporthe* EB4 might promote tomato plant growth and confer tolerance to drought stress by improving soil uptake of mineral nutrients, chlorophyll content, leaf photosynthesis, and K–mediated stomatal dynamics (**Figure 7**).

In response to ROS production caused by drought stress, plants have developed an intricate antioxidant defense network composed of enzymatic and non–enzymatic antioxidants that scavenge ROS and maintain cellular redox homeostasis (Ahmad et al., 2010; Muhammad et al., 2021). In our study, APX and CAT, both H_2_O_2_ scavenging enzymes, varied their activities under drought stress regardless of inoculation treatment, although in different ways. The activity of CAT increased under drought stress, implying that H_2_O_2_ accumulated in the plant cells, and this activity was notably higher in inoculated plants under drought. We thus propose that *Diaporthe* EB4 could similarly confer tolerance to drought through an induced mechanism, in which the activity of some antioxidant enzymes like CAT could be modulated.

Intriguingly, under drought stress the APX activity decreased, while the DHAR activity increased. Both APX and DHAR belong to the ascorbate-glutathione cycle. The decrease in APX activity is probably due to a lower content of ascorbate in leaf cells, which is consistent with the lower growth of tomato plants under drought stress and the role of ascorbate in cell expansion and cell division (Foyer, 2018). APX was not significantly affected by *Diaporthe* inoculation. However, the significant increase in DHAR activity in inoculated plants under drought suggested that cellular ascorbate regeneration was better in the presence of *Diaporthe* EB4, although the content of ascorbate in inoculated plants probably did not reach levels similar to those under well-watered conditions on the basis of plant biomass. Altogether, *Diaporthe* EB4 could improve the enzymatic antioxidant response of tomato plants and confer protection against ROS generation during drought stress (**Figure 7**).

Additionally, fungal endophytes can induce the formation non–enzymatic antioxidant metabolites such as phenolic compounds (White and Torres, 2010; Bacon and White, 2016; Varela et al., 2016). In our previous studies, *Diaporthe* EB4 did not enhance the total phenolic content in grasses under control or salt stress conditions (Vázquez de Aldana et al., 2021; Toghueo et al., 2022). In the present study, we obtained rather similar results and *Diaporthe* seemed to induce a decline in the non–enzymatic antioxidant capacity under drought stress and to have no significant effect on the total phenolic content.

Osmotic adjustment through the accumulation of solutes such as proline is an important mechanism of plant adaptation to salinity and drought (Munns, 2011; Kaur and Asthir, 2015). In fact, an enhanced accumulation of proline due to inoculation with *Diaporthe* EB4 also occurred in plants of tritordeum under salt stress (Toghueo et al., 2022). In addition to its role as osmolyte, proline interacts with protein and membranes stabilizing their structures and activities (Farooq et al., 2009; Zivcak et al., 2016) and deters oxidative damage through scavenging of ROS, such as hydroxyl radicals formed during H_2_O_2_ decomposition within the Fenton reaction (Das and Roychoudhury, 2014). In this study, the highest proline accumulation was detected in inoculated plants under drought, a result in line with previous studies in which fungal endophytes like *Penicillium* sp., *Trichoderma harzianum*, DSE, or *Piriformospora indica* conferred drought tolerance to several crops and increased accumulation of proline as osmoprotectant (Molina-Montenegro et al., 2016; Alwhibi et al., 2017; Valli and Muthukumar, 2018; Swetha and Padmavathi, 2020). This accumulation of proline did not seem to notably reduce the loss of water molecules on the basis of the stomatal conductance. This led us to propose, together with its role as an osmoprotectant and ROS scavenger, that proline is also a source of reducing power (NADPH) that plants can use to produce ATP in the dark, showing an oscillating day/night content pattern (Signorelli, 2016) as they also use the accumulation of osmoprotectant sugars under drought stress to produce cell energy when the stress ceases (Ghaffari et al., 2019).

In conclusion, this study shows the capacity of *Diaporthe atlantica*, a fungus symbiotic with plants adapted to a saline environment, to promote growth and adaptation to drought stress on tomato. *Diaporthe* played a positive role in the modulation of tomato responses to drought stress through the combination of various processes. *Diaporthe* could confer drought stress tolerance to tomato by improving soil uptake of mineral nutrients, chlorophyll content, leaf photosynthesis and K-mediated stomatal dynamics. In addition, *Diaporthe* could improve the enzymatic antioxidant response of tomato, through an induced mechanism in which the activity of some enzymes like CAT could be modulated and confer protection against ROS generation during drought stress. An enhanced accumulation of proline could also play an important role in the response of plants to water stress, acting as osmoprotectant, ROS scavenger, and a source of reducing power to produce energy. In general, these results indicate that symbiotic fungi can enhance tomato tolerance to drought stress.

## Data availability statement

The raw data supporting the conclusions of this article will be made available by the authors, without undue reservation.

## Author contributions

EP performed experiments and analyses. All authors designed the experiments, worked on the analyses of data, wrote the manuscript, and approved the submitted version.
